# Respiratory syncytial virus subtypes in children with bronchiolitis: does it correlate with clinical severity?

**DOI:** 10.1186/s12879-024-09129-y

**Published:** 2024-02-26

**Authors:** Shuo Yang, Sukun Lu, Yakun Wang, Yinghui Guo, Zhuang Zhang, Weijian Wang, Le Wang

**Affiliations:** 1 Institute of Pediatric Research, Children’s Hospital of Hebei, 133 Jianhua South Street, 050031 Shijiazhuang, Hebei Province China; 2 Ningbo HEALTH Gene Technologies Co., Ltd, 315000 Ningbo, China

**Keywords:** Bronchiolitis, Children, RSV subtypes, Outcome

## Abstract

**Background:**

In this retrospective study, we aimed to evaluate the factors associated with the severity of respiratory syncytial virus (RSV) bronchiolitis in children aged under 2 years who were admitted to the Children’s Hospital of Hebei between June 2018 and January 2019.

**Methods:**

Sputum samples positive for RSV via multiplex PCR were subtyped using real-time PCR. Data collected included risk factors for disease severity, demographics, microbiology, and outcomes.

**Results:**

Of the 82 children with RSV bronchiolitis, 79 were treated and discharged with improvement, while 3 died. All three patients had underlying medical conditions, including complex congenital heart disease and severe combined immunodeficiency. Further, disease severity was associated with preexisting underlying disease, fever duration, and bacterial co-infection, but not with the RSV subtype.

**Conclusions:**

Our findings suggest that an appropriate therapeutic regimen should include the detection of bacterial co-infections and the identification of underlying diseases for the effective management of severe RSV bronchiolitis.

## Introduction

RSV is a major pathogen that often causes outbreaks during the cold season [1]. By 2 years of age, more than 90% of children have serological evidence of RSV infection [2]. RSV is commonly present in young infants and children with bronchiolitis, which is a lower respiratory tract infection (LRTI) with small airway obstruction, and can rarely progresses to pneumonia, respiratory failure, apnea, and death [3]. RSV causes approximately 33 million LRT illnesses, 3 million hospitalizations, and up to 199,000 childhood deaths worldwide, with RSV bronchiolitis accounting for the largest proportion [4, 5]. Many countries have updated their bronchiolitis guidelines [6–9] to define early-life severe RSV bronchiolitis and to prevent its potential long-term poor prognosis, including recurrent episodes of wheezing and asthma after recovery from bronchiolitis or pneumonia [10, 11].

Despite intensive research, safe and effective vaccines for preventing RSV bronchiolitis remain elusive [12]. Currently, only one approved antiviral treatment for RSV is available; however, its use is limited by questionable efficacy, side effects, cost, and it is not accessible in our country China [13]. The widespread administration of prophylactic drugs emphasizes the need for active surveillance of RSV subtypes and the timely detection of viral mutants. Only two subtypes (RSV-A and RSV-B) are prevalent, and the clinical impact of viral factors associated with RSV remains controversial. Some studies have reported no differences between the two subtypes [14, 15], while others have shown that RSV-A has a worse [15, 16] or better prognosis than RSV-B [17, 18]. In the absence of vaccine coverage and due to the inaccessibility of antiviral medicines, understanding the differences in clinical symptoms severity and outcomes caused by different subtypes could help select appropriate therapeutic regimens and formulate vaccine development strategies. In this study, we aimed to investigate the subtype-specific severity of RSV infection in paediatric bronchiolitis and identify factors that may be associated with disease severity.

## Methods

### Ethics approval

This study was approved by the Medical Research Ethics Committee of the Children’s Hospital of Hebei (CHH) in accordance with the principles of the Declaration of Helsinki and the Code of Ethics of the World Medical Association. As this was a retrospective study that poses no risk of harm to the subjects, and all patients were de-identified, informed consent was waived by the committee.

### Study subjects

Children (aged < 2 years) admitted to our hospital between June 2018 and January 2019 with a discharge diagnosis of RSV bronchiolitis were included in this study. Demographic data, clinical characteristics, underlying diseases, laboratory test results and treatment outcomes of these patients were retrieved and retrospectively analyzed. Patients who underwent repeat testing within 14 days were excluded from the study.

### Pathogen detection

A multiplex PCR-based platform (i.e., GenomeLab system, Beckman countler, USA) was used to simultaneously detect RSV and 10 other pathogens: influenza A virus, influenza B virus, adenovirus, parainfluenza virus, human rhinovirus, human metapneumovirus, human bocavirus, human coronavirus, *Chlamydia pneumoniae*, and *Mycoplasma pneumoniae*. Multiplex PCR was performed as previously described [19]. RSV-positive samples were tested for RSV-A and RSV-B using a real-time RT-PCR (RT-qPCR) assay (Liferiver Biotechnology Co. Ltd) according to the manufacturer’s recommendations. Bacterial and fungal cultures from induced sputum (IS) and blood samples were isolated according to the protocols developed in our diagnostic laboratory.

To obtain an IS sample, a sterile negative-pressure suction catheter was used by a skilled nurse to stimulate the throat and induce coughing. Evidence of bacterial co-infection was proven using blood cultures from sterile sites or IS from non-sterile sites. For positively induced sputum, if the clinician judged that it was clinically significant and an appropriate antibiotic treatment was administered, we also regarded it as a bacterial co-infection.

### Disease severity and complications

Disease diagnosis was performed according to the “Clinical Practice Guideline: The Diagnosis, Management, and Prevention of Bronchiolitis” [8]. Meanwhile, according to the Chinese guidelines on bronchiolitis, severe disease was defined as the presence of one or more of the following manifestations: (1) increasing irritability and/or lethargy, fatigue; (2) marked increase in respiratory rate; (3) marked chest wall retractions, marked tracheal tugging; (4) marked nasal flaring; (5) O_2_ saturation less than 88% (in room air) and hypoxemia, which may not be corrected by O_2_; (6) increasingly frequent or prolonged apnea; or (7) reluctance or inability to feed [7]. Complications in the cardiovascular system involvement included heart failure, abnormal cardiac enzyme profiles, and right heart failure; while those in the gastrointestinal system included diarrhea, vomiting, and gastrointestinal bleeding.

### Treatment and outcomes

All patients were treated according to the “Clinical Practice Guidelines: The Diagnosis, Management, and Prevention of Bronchiolitis” [8]. The outcomes recorded included recovery and discharge, transfer to a community hospital, or death.

### Statistical analyses

Normality of the data was determined using the Shapiro-Wilk test. Non-normal data are presented as medians (first and third quantiles) and analyzed using the Mann-Whitney test. Categorical data were analyzed using the chi-squared or Fisher’s exact tests. Logistic regression analysis was performed to select the variables associated with severe RSV bronchiolitis. Statistical analyses were performed using SPSS version 23.0. A *p* <.05 was considered statistically significant.

## Results

### Study Population and RSV subtyping

**Fig. 1:**
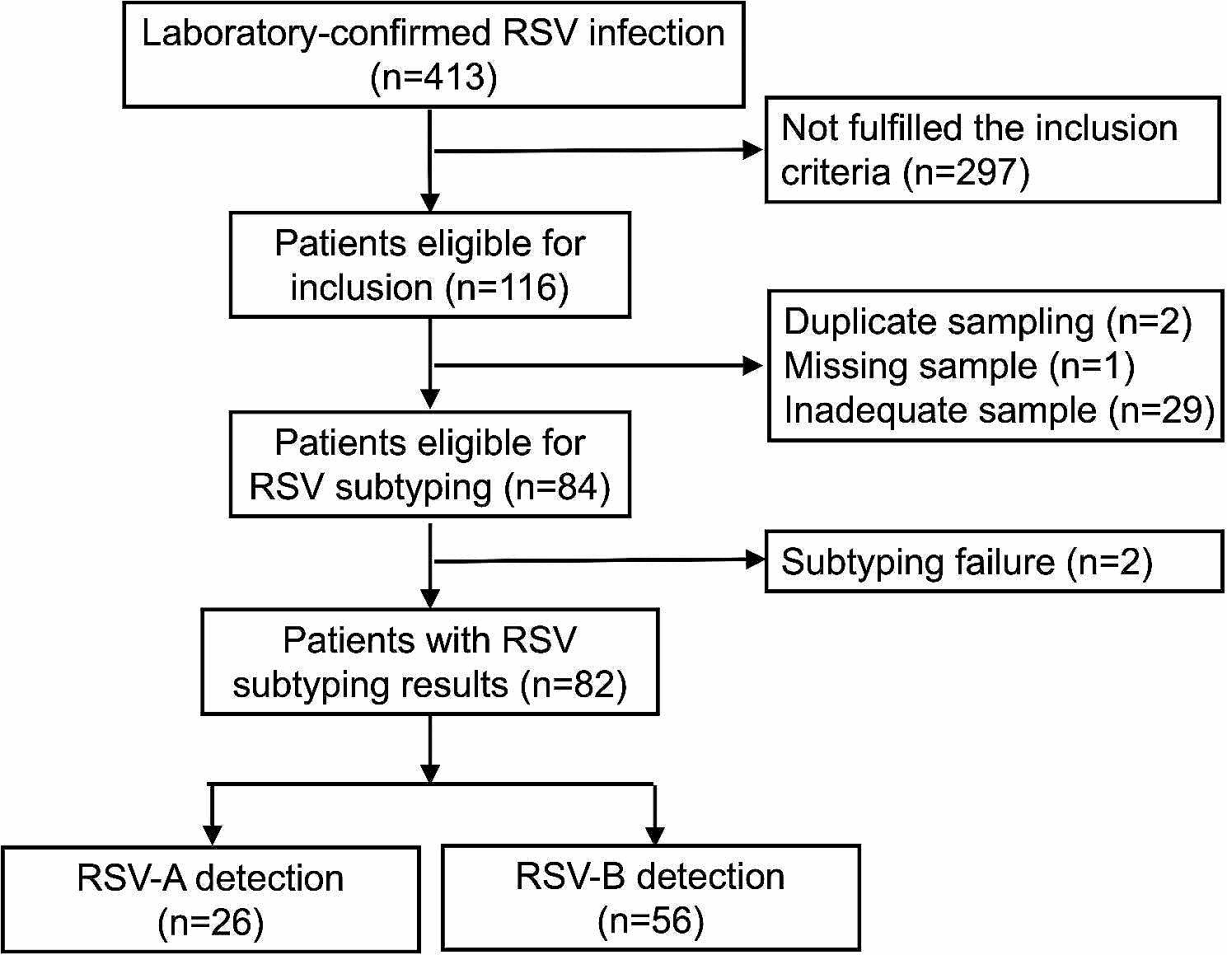
Flow-chart of patient enrollment

During the observation period of this study, 413 children with laboratory-confirmed RSV infections were hospitalized for treatment, among which 116 patients met the inclusion criteria. Of the 116 patients, 32 had missing, insufficient, or duplicate samples; while 2 were unsuccessfully subtyped. Finally, 82 IS samples were successfully subtyped (Fig. 1). The median age of the patients was 3 months (IQR: 2–10), and 32% (26/82) were subtyped with RSV-A and 68% (56/82) with RSV-B. Except for hospital stay, which was longer in children with RSV-A than in those with RSV-B (*p* =.044), no significant differences in disease severity, laboratory test results, or clinical features were observed between the two RSV subtypes (Table 1).

**Table 1:** Demographic, laboratory data and outcomes of children with RSV bronchiolitis

Characteristics		RSV-A *n* = 26	RSV-B *n* = 56	P value
Demographics	Age, months	3.5 (2, 10)	3 (2, 10)	0.680
	Gender (male)	14 (54%)	35 (62%)	0.457
Clinical features	Underlying diseases	8 (31%)	17 (30%)	0.970
	Extrapulmonary complications*	10 (39%)	23 (41%)	0.823
	Disease day before admission	4 (3, 8)	5 (3, 7)	0.500
	Febrile day before admission	1 (0, 3)	1 (0, 3)	0.622
	Highest temperature (℃)	38 (37, 39)	38 (37, 38.9)	0.971
Laboratory testing results	WBC (10^9/L)	9 (6.8, 12.9)	9 (7, 12)	0.823
	Neutrophils%	32.5 (19.6, 60.2)	34 (22, 52)	0.762
	Lymphocytes%	53 (27, 66)	57 (38, 64)	0.931
	Monocytes%	8 (5.5, 11)	8 (5.7, 10)	0.598
	CRP (mg/L)	1 (1, 10.9)	1 (1, 7.2)	0.390
	CD3 + cell%	59 (58, 62)	59 (55, 65)	0.976
	CD4 + cell%	37 (32, 37)	37 (35, 41)	0.167
	CD8 + cell%	20 (17, 22)	19 (16, 21)	0.243
	DNT cell%	2.9 (1.9, 3.1)	2 (1.4, 2.9)	0.072
	CK (U/L)	84 (68, 165)	92 (67, 121)	0.932
	CK-MB (ug/L)	3 (1.5, 6.1)	3.8 (2.1, 4.6)	0.580
	LDH (U/L)	327 (259, 414)	287 (256, 317)	0.109
	HBDH (U/L)	256 (216, 310)	236 (213, 256)	0.209
	Bacterial coinfection	5 (19%)	6 (11%)	0.292
Outcomes	Severe/moderate/mild	13/3/10	21/2/33	0.139
	PICU admission	18 (69%)	26 (46%)	0.054
	PICU stay	1 (1,2)	0 (0,1)	0.300
	Hospital stay	8 (7,11)	7 (6,8)	0.044
	Death	2 (7.7%)	1 (1.8%)	0.235

*Extrapulmonary complications included 21 children with cardiovascular and 19 children with digestive system involvement. Nine of these children had both cardiovascular and digestive complications, making a total of 33 children with all complications

Abbreviations: WBC, white blood cell; CRP, C-reactive protein; DNT, double negative T cell; CK, creatine kinase; CK-MB, creatine kinase isoenzymes MB; LDH, lactate dehydrogenase; HBDH, hydroxybutyrate dehydrogenase

### Clinical features and outcomes

A total of 34 patients had severe bronchiolitis, while 48 as mild (43 patients) to moderate disease (5 patients). The demographic and clinical characteristics of the two groups are shown in Table 2. Patients in the severe group were younger (2 vs. 5 months of age, *p* =.004) and had a significantly shorter duration of fever (*p* =.018) and lower peak fever before admission (*p* =.033), compared to those in the mild-to-moderate group. A significantly greater proportion of the severe group had underlying diseases (44% vs. 21%, *p* =.024) and extrapulmonary complications (56% vs. 29%, *p* =.015), compared to the mild-to-moderate group (Table 2).

**Table 2:** Demographic, laboratory, clinical data, and treatment outcomes between severe and mild to moderate patients

		Severe	Mild to moderate	*P* value
		*n* = 34	*n* = 48	
Demographics	Age, median months (IQR)	2 (1, 4)	5 (2, 16)	0.004
	Gender (male)	20 (59%)	29 (60%)	0.885
Clinical features	Underlying diseases	15 (44%)	10 (21%)	0.024
	Peak fever (℃)	37.3 (37, 38.6)	38.2 (37, 39)	0.033
	Febrile duration	0 (0, 2)	1 (0, 4)	0.018
Laboratory testing results	WBC (10^9/L)	8.4 (6.8, 11.7)	9.9 (7.0, 12.7)	0.423
	Neutrophil%	31 (21, 59)	36 (22, 49)	0.826
	Lymphocyte%	57 (34, 65)	56 (37, 65)	0.872
	Monocyte%	9 (5, 10)	8 (7, 9)	0.856
	CRP (mg/L)	1 (1, 10.6)	1 (1, 6.8)	0.685
	CD4 + cell%	37 (36, 38)	37 (35, 41)	0.603
	CD8 + cell%	20 (17, 21)	20 (16, 23)	0.817
	CD19 + cell%	36 (31, 40)	26 (34, 37)	0.097
	DNT cell%	2 (1.0, 3.0)	2.4 (1.9, 3.0)	0.038
	CK (U/L)	98 (65, 132)	85 (68, 118)	0.944
	CK-MB (ug/L)	4.1 (2.4, 7.9)	2.9 (1.7, 4.7)	0.057
	LDH (U/L)	285 (259, 381)	303 (256, 330)	0.936
	HBDH (U/L)	231 (208, 298)	248 (219, 269)	0.675
Outcomes	Extrapulmonary complications	19 (56%)	14 (29%)	0.015
	Hospital stay	8 (7,11)	7 (6,8)	0.016
	PICU admission	32 (94%)	12 (25%)	< 0.001
	PICU stay	1 (1,2)	0 (0,1)	< 0.001
	Death	3 (9%)	0	0.134

Abbreviations: WBC, white blood cell; CRP, C-reactive protein; DNT, double negative T cell; CK, creatine kinase; CK-MB, creatine kinase isoenzymes MB; LDH, lactate dehydrogenase; HBDH, hydroxybutyrate dehydrogenase

All children in the mild-to-moderate group recovered or improved, whereas three children aged 2–4 months in the severe group died (Table 3). All three patients had underlying medical conditions: two had complex congenital heart disease and one had combined immunodeficiency syndrome (CIS).

**Table 3:** Clinical features of three death cases

Case	Gender	Age (Month)	Preterm birth	RSV subtype	Bacterial co-infection	Underlying Disease	ICU admission	Invasive mechanical ventilation	Extra pulmonary complications
50	Female	3	No	A	No	CHD	Yes	Yes	Digestive system
55	Male	4	Yes	B	*A. baumannii*	CIS	Yes	Yes	No
77	Female	2	Yes	A	*P. aeruginosa*	CHD	Yes	Yes	Cardiovascular system

Abbreviations: CHD, congenital heart disease; CIS, combined immunodeficiency syndrome

### Laboratory testing results

Results showed that, except for a lower percentage of double negative T cells (DNT, CD3 + CD4-CD8-) in patients with severe bronchiolitis, there were no significant differences in other markers, including white blood cell count, C-reactive protein, and lactate dehydrogenase (LDH), compared to the mild-to-moderate group (Table 2).

### Bacterial co-infection

Of the 82 subtyped samples, 20 (24.4%) were detected simultaneously with other respiratory pathogens, of which 11 (32.3%) were detected in the severe group and 9 (18.8%) in the mild-to-moderate group (*p* =.158, Table 4). Compared to the mild-to-moderate group. the percentage of patients with bacterial co-infection was significantly higher in the severe group (26% vs. 4%, *p* =.010), whereas no significant differences in the rates of viral or atypical bacterial co-infections were observed (all *p* >.05, Table 4). Further, no association was observed between the RSV subtype and severity, even when cases with co-infections were excluded (*p* =.742; data not shown).

**Table 4:** Coinfections detected in severe and mild to moderate cases

Etiological agents	Total	Severe	Mild to moderate	P value
	*n* = 82	*n* = 34	*n* = 48	
RSV mono-detection	62	23	39	**0.158**
RSV co-detection	**20**	**11**	**9**	
**Virus, n**	**7**	**2**	**5**	**0.261**
HRV	1	0	1	
HBoV	1	1	0	
HMPV	1	0	1	
FluA	3	1	2	
FluA + HRV	1	0	1	
**Bacteria, n**	**11**	**9**	**2**	**0.010**
*S. aureus*	2	2	0	
*K. pneumoniae*	1	1	0	
*S. pneumoniae*	1	1	0	
*H. influenzae*	1	1	0	
*P. aeruginosa*	1	1	0	
*A. baumannii*	1	1	0	
*S. marcescens*	1	0	1	
*S. marcescens + C. pneumoniae*	1	1	0	
*S. aureus +* HRV + FluA	1	0	1	
*K. pneumoniae* + *C.pneumoniae +* HRV	1	1	0	
***C.pneumoniae***, **n**	**2**	**0**	**2**	**0.509**
*C. pneumoniae +* HRV	1	0	1	
*C. pneumoniae*	1	0	1	

Abbreviations: HRV, human rhinovirus; HBoV, human bocavirus; HMPV, human metapneumovirus; Flu A, Influenza A; *S. aureus, Staphylococcus aureus*; *K. pneumoniae, Klebsiella pneumoniae; S. pneumoniae, Streptococcus pneumoniae; H. influenza, Haemophilus influenzae; P. aeruginosa Pseudomonas aeruginosa; A. baumannii, Acinetobacter baumannii; S. marcescens, Serratia marcescens*; C. *pneumoniae, Chlamydia pneumoniae*

### Multiple logistic regression

Non-conditional multiple logistic regression analyses were performed to assess the markers of severe RSV bronchiolitis. Fever duration was a protective factor (OR [odds ratio] = 0.693, *p* =.010), whereas bacterial co-infection possessed significantly greater predictive values as an independent risk factor for severe RSV bronchiolitis with an OR value of 16.365 (95% CI 2.388, 112.159, *p* =.004) (Table 5).

**Table 5:** Stepwise logistic regression analysis for the related factors predicting the severe RSV bronchiolitis

Variables	B	S.E.	Wald	OR	95% CI		P value
					Lower	Upper	
Age	-0.028	0.046	0.37	0.972	0.889	1.064	0.543
Bacterial co-infection	2.795	0.982	8.101	16.365	2.388	112.159	0.004
Febrile duration	-0.366	0.142	6.619	0.693	0.525	0.916	0.010
Peak fever	-0.348	0.384	0.822	0.706	0.333	1.498	0.365
DNT cells	-0.118	0.216	0.298	0.889	0.583	1.356	0.585
Underlying disease	0.429	0.570	0.567	1.536	0.502	4.698	0.452

## Discussions

RSV is the most common cause of bronchiolitis that leads to hospitalisation in young children [20, 21]. RSV outbreaks are common in autumn and winter, and the severity and serotypes of RSV infection in a given country vary annually [4]. In China, subtype A was predominant from 2011 to 2017 [22]. After 2018, the prevalence of RSV-B exceeded that of subtype A in China [23], Japan [24] and India [25]. The present study found that, subtype B was twice as prevalent as subtype A in young children with bronchiolitis. After COVID-19, RSV-A outbreak was predominant [26, 27]. Nevertheless, the impact of molecular epidemiology on the clinical course of the disease remains controversial [4, 28, 29]. Based on the bronchiolitis guidelines, we investigated disease severity and its correlation in our patients with RSV bronchiolitis. We found that: (1) a greater proportion of severely ill children had shorter fever duration and bacterial co-infections; (2) RSV subtypes were not associated with disease severity.

RSV can cause complications in other organ system in children [30–32]. Our results showed that, 25.6% of children with RSV bronchiolitis developed cardiovascular complications, ranging from myocardial injury to heart failure. A previous study on cardiovascular involvement in hospitalized children with RSV infection reported that approximately 76.5% of otherwise healthy infants with RSV bronchiolitis showed sinoatrial blocks and transient rhythm alterations [30]. A study conducted in Japan found that 50% (9/18) of children with RSV infection had myocardial damage, 38.8% had conduction system disturbances and 16.6% had tachycardia [33]. Thorburn et al. reported that, 20% (7/34) of children with severe RSV bronchiolitis admitted to a tertiary pediatric-intensive unit in the UK had reduced right ventricular function [34].

Furthermore, the extrapulmonary complications have been described also in the digestive, neurological, and other organ systems [32]. In the present study, 23.1% (19/82) of patients had gastrointestinal complications. In the USA, a previous case series reported that four infants with severe RSV bronchiolitis developed necrotizing enterocolitis shortly after admission [35]. In addition, 87 unique studies from 26 countries described a spectrum of RSV-associated severe acute neurological syndromes, including proven encephalitis, acute encephalopathy, complex seizures, hyponatremic seizures, and immune-mediated disorders [36]. These data suggest that RSV-associated extrapulmonary complications are common in children and can lead to high morbidity and mortality. Thus, management of such complications should be a critical part of the therapeutic regimen.

Persistent fever can worsen infectious diseases, and some studies reported a higher mortality rate for fever lasting more than 5 days [37]. On the contrary, it has also been reported that fever may induce the expression of heat shock proteins that protect host cells and regulate immune responses [38]. In the present work, the median duration of fever in severely ill children was 0 days, which was significantly shorter than 1 day in patients with mild to moderate bronchiolitis. We found this relatively short fever course to be a protective factor against severe bronchiolitis. On the other hand, aggressive hypothermia treatment does not alleviate disease progression. Schulman et al. reported that aggressive fever suppression (administration of paracetamol above 38.5 °C and application of a cooling blanket above 39.5 °C) was associated with significantly higher mortality compared to permissive suppression (the same interventions above 40 °C, 15.9% versus 2.6%, *p* =.06, Fisher’s exact test) [39]. These data revealed that fever duration may serve as a disease severity marker, which requires further study.

Lymphopenia and its association with disease severity have been reported in children with RSV infections [40, 41]. A histopathological study of children with fatal RSV infection showed that double-negative T (DNT) cells infiltrated the bronchial and pulmonary arterioles, and promoted the formation of fibrin, mucus, and edema [42]. The number of circulating DNT cells in peripheral blood mononuclear cells (PBMCs) was lower in patients with chronic autoimmune diseases than in healthy controls [43]. A reduced number of DNT cells may lead to a loss of immune regulation, thereby breaking immune tolerance and promoting pathogenesis [44]. In the present study, a substantially lower percentage of DNT cells was observed in the severe group compared to that in the mild-to-moderate group, suggesting that a decrease in DNT cells may reflect the disease severity.

Bacterial co-infection with RSV in severely ill children with RSV has been described previously [45, 46]. In a study aimed at comparing outcomes between RSV with and without bacterial co-infection in children without underlying diseases, Lin et al. showed that children with co-infections required more intensive care and a longer hospital stays [45]. In another study, the presence of bacterial co-infection was significantly associated with the development of acute respiratory distress syndrome (OR = 1.9) in children with RSV infection [47]. Similar to the findings of Ghazali et al. [46], we observed a significantly higher rate of bacterial co-infections in the severely ill children. Because some studies only tested for RSV and did not consider other viral or bacterial infections, confounding effects may have been omitted or have led to ascertainment bias. Our results suggest that the early recognition of bacterial infections and prompt, effective antibiotic treatment of suspected severe cases are important for preventing disease progression.

Our study had several limitations. First, this was a retrospective study involving a single center. Second, the sample size was relatively small, including only 26 patients with severe disease. Third, not all patients with RSV bronchiolitis underwent RSV subtyping. Fourth, we observed that the duration of fever was significantly shorter in the severe group, which requires further investigation of the relationship between disease severity and fever. Furthermore, bronchiolitis was screened in children using a laboratory molecular diagnosis for RSV infection. Although some studies have shown that the vast majority of children with bronchiolitis are infected with RSV, bronchiolitis due to infection by other pathogens may have also been neglected.

## Conclusion

In conclusion, a high proportion of children with severe RSV bronchiolitis have underlying diseases and extrapulmonary complications. Children with severe disease may also have a higher incidence of bacterial co-infection and lower levels of adaptive immunity. Therefore, treatment regimens for severe bronchiolitis should include organ-specific supportive care, antibiotics for bacterial co-infections, and immune-boosting treatments.

## Data Availability

The datasets used and/or analysed during the current study are available from the corresponding author on reasonable request.

## Abbreviations

RSV: Respiratory syncytial virus
LRT: Lower respiratory tract
CHH: Children’s Hospital of Hebei
HRV: human rhinovirus
PCR: Polymerase chain reaction
PIV: Human parainfluenza virus
HCoV: Human coronavirus
ADV: Adenovirus
HBoV: Human bocavirus
FluA: Influenza A virus
FluB: Influenza B virus
HMPV: human metapneumovirus
C.: pneumoniae Chlamydia pneumoniae
S.: pneumoniae Streptococcus pneumoniae
H.: influenzae Haemophilus influenza
S.: aureus Staphylococcus aureus
P.: aeruginosa Pseudomonas aeruginosa
A.: baumannii Acinetobacter baumannii
S.: marcescens Serratia marcescens
